# Combined small and large magnetic nanoparticle extraction and concentration from biofluids for non-toxic detection of biomarkers†

**DOI:** 10.1039/d2sd00078d

**Published:** 2022-06-09

**Authors:** Anatoliy S. Lapchuk, Ivan V. Gorbov, Alexander V. Prygun, Iryna V. Balagura, Yevhenii M. Morozov

**Affiliations:** Department of Optical Engineering, Institute for Information Recording of NAS of Ukraine 03113 Kyiv Ukraine alapchuk@yahoo.com; Biosensor Technologies, AIT-Austrian Institute of Technology 3430 Tulln Austria Yevhenii.Morozov@ait.ac.at

## Abstract

We propose a novel non-toxic method of diagnostic biomarker extraction and concentration from biofluids. The method is based on the usage of (1) magnetic nanoparticles of a few nanometres in size bearing molecular traps for biomarkers on their surface and (2) additional larger (several tens of nanometres) magnetic nanoparticles for catching smaller magnetic nanoparticles in a strong magnetic field gradient with their consequent concentration into the detection area. It is shown that the interference of an external permanent gradient magnetic field with the magnetic field of large magnetic nanoparticles allows one to catch small magnetic nanoparticles from their trajectories in a fluid at a distance around ten radii of the large nanoparticles. Theoretical analysis and mathematical simulation show the validity of the proposed non-toxic method for fast and robust biomarker extraction and concentration for increasing the sensitivity of biomarker detection. We believe that the results presented herein can serve as a starting point in the development of a new subclass of biosensors and a human body diagnostic approach with enhanced sensitivity and selectivity.

## Abstract 2

Sketch of the concentration of small and large magnetic nanoparticles in biofluid to the area of biomarker detection for enhancing the method sensitivity.

## Graphical abstract

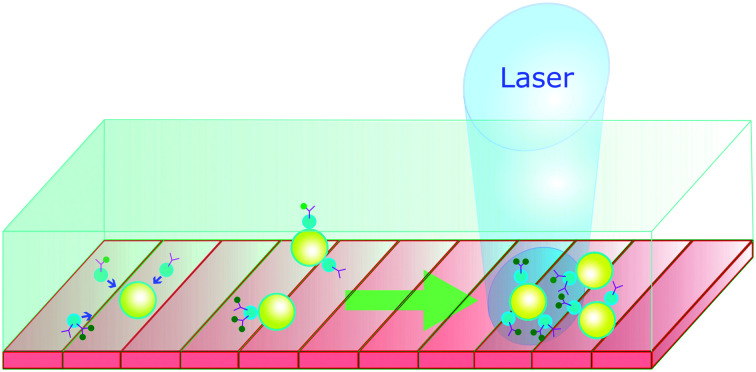

## Introduction

The early detection of pathological development of diseases such as cancer,^1^ Alzheimer's disease,^2^ or cardiovascular disease,^3,4^ to name a few, is very important to avoid severe consequences and death. One of the methods allowing recognition of a disease at its early stage of development is detection of the pathological biomarkers and measurement of their concentration.^5–11^ However, at the early stage, marker concentration is deficient, and therefore, a significant signal and signal-to-noise ratio amplification is needed for robust detection.^12,13^ The surface plasmon resonance is often used to get substantial signal enhancement.^14–17^ However, in order to get a strong plasmon-enhanced effect, biomarkers should be fixed and concentrated near the plasmonic surface using a molecular trap.^17^ It is also possible to increase the signal by concentrating biomarkers at a specific volume area by means of, *e.g.*, magnetic forces^18–20^ or plasmonic interactions.^21^ Organism biofluids are commonly used for biomarker catching with the help of molecular traps at a specially developed surface. An organism biofluid can be blood,^22–24^ urine,^24–26^ saliva,^24^ or even sweat.^27^ In order to have a higher probability of biomarkers gathering at a specific area of the surface bearing molecular traps, the ratio of surface to volume should be as large as possible, and a biofluid should be actively moving over the surface. The smallest nanoparticles match the above-mentioned criteria well, since they have the highest surface-to-volume ratio and have the largest velocity due to the kinetic motion. Additionally, only a small amount of a fluid can be extracted from an organism relative to the total fluid contained in it. Therefore, to achieve the most sensitive biomarker detection method, one should use the maximum possible amount of a biofluid, and it is only possible to achieve that by injecting nanoparticles into the blood circulatory system of a living organism. It is well known that only nanoparticles of the size of 2–5 nm can be effectively and in a fast manner extracted through the kidneys by the urine stream with the smallest toxicity level to the organism.^28–31^ This type of nanoparticle with attached biological traps possesses the ability to circulate through the blood and urine streams all over the organism's organs. Larger nanoparticles circulate much longer in the human organism and are concentrated in Kupffer cells in the liver, and to a lesser extent are captured by macrophages in the spleen and other organs, followed by the degradation and excretion from the body.^32–35^ This leads to intoxication of the liver and other organs. In fact, larger nanoparticles can be obtained intact only through blood sampling. Therefore, for larger molecule biomarkers, the blood should be used for disease marker detection.

Magnetic force can be used to the concentrate extracted from biofluid nanoparticles for strong signal enhancement by using small non-toxic magnetic nanoparticles (mNPs).^36^ However, small mNPs of a few nanometres in size are very difficult to manipulate and concentrate by any forces due to the domination of the viscosity force.^37^ This strongly limits the efficiency of concentration and, hence, biomarker detection sensitivity, as well as the time required for the mNP concentration and data acquisition.

**Fig. 1:**
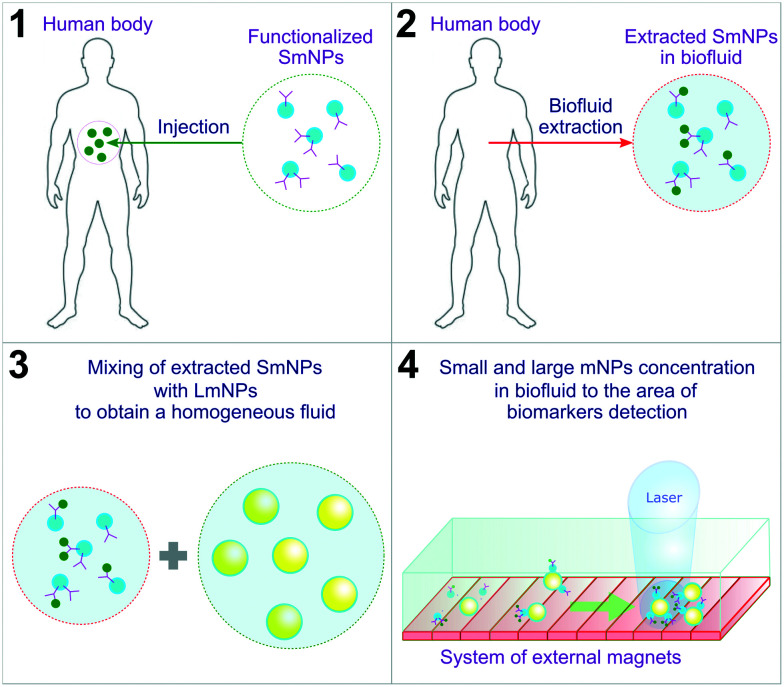
Overview of the proposed method steps. SmNPs – small magnetic nanoparticles; LmNPs – large magnetic nanoparticles; mNPs – magnetic nanoparticles.

In this manuscript, we propose a diagnostic method based on the use of “small” mNPs (SmNPs) of a few nanometres in size, which are potentially capable of bearing biomarker traps (such as antibodies) on their surface (see Fig. 1 with an overview of the method steps).

An external system of magnets concentrates functionalized SmNPs in a biofluid extracted from the human body to increase the signal and signal-to-noise ratios. Besides, in the method, it is proposed to use additional “large” mNPs (LmNPs) with a size of several tens of nanometres. After the extraction of the biofluid with SmNPs and mixing the fluid with LmNPs (aiming to get a homogeneous distribution of LmNPs), a thin flow of the obtained fluid is placed under a strong gradient magnetic field. LmNPs move relatively fast in the magnetic field gradient to the point of the field maximum and consequently concentrate at that point. Besides, LmNPs in an external magnetic field can be considered as secondary magnets creating their own magnetic field in the surroundings. In general, the force applied to mNPs in the magnetic field is proportional to the magnetic field gradient. The magnetic field gradient is, in turn, inversely proportional to the linear size of the magnets. Therefore, due to the ratio of their sizes – millimetres for the primary magnets (such as the Halbach array of permanent magnets^38^) and tens of nanometres for the secondary ones (large mNPs) – large mNPs create a magnetic force on small mNPs, which is stronger than the external magnetic field force induced by the permanent magnets used for magnetic particle concentration. In other words, this means an increase of tens of thousands of times of magnetic force applied to SmNPs near the LmNPs. This increase is significant enough for the attraction and “adsorption” of the SmNPs on the surface of the LmNPs and their consequent drift to the magnetic field maximum where biomarkers can be eventually detected by, *e.g.*, optical means. To prove the reliability of the proposed method, we designed and analysed a simple mathematical model of interaction of large and small mNPs in an external magnetic field. The results presented in this paper aim to be a starting point of the development of a new subclass of biosensors and a human body diagnostic approach.

## Mathematical model of the two-component colloidal solution of large and small mNPs in an external gradient magnetic field

**Fig. 2:**
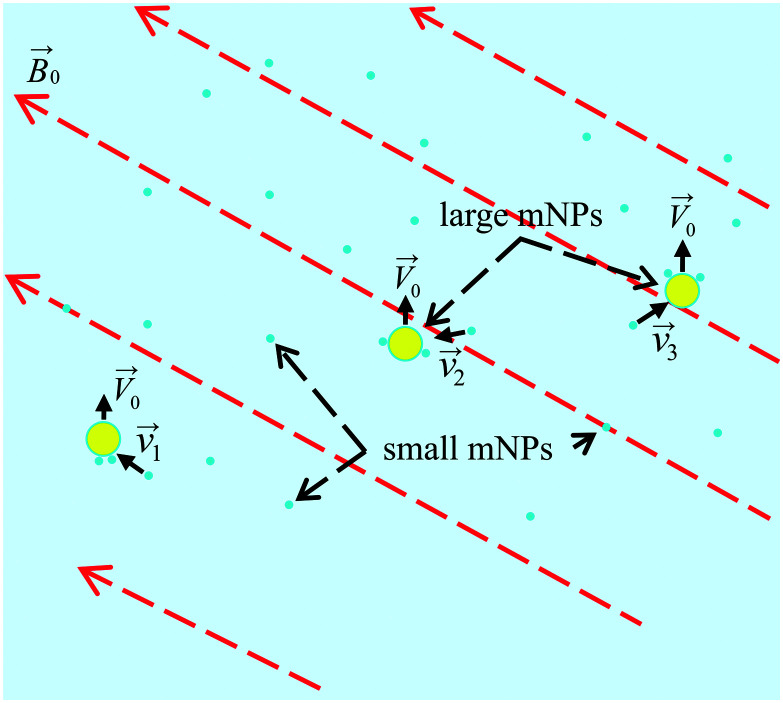
Scheme of the LmNP motion (with a stochastic coagulation of SmNPs on their surface) through a volume of the colloidal solution under the action of a gradient magnetic field.

In order to develop and optimise the proposed method, it is crucial to simulate the interaction and relative motion of two sorts (small and large) of mNPs in a colloidal solution (biofluid) when large mNPs pass the area near the small ones. In Fig. 2, a schematic of the colloidal solution with two sorts of mNPs of different sizes is shown. Nanoparticles are considered to be made of a ferrimagnetic material (such as magnetite (Fe_3_O_4_) or maghemite (γ-Fe_2_O_3_)) with a fixed relative magnetic permeability *μ*_NP_ and a fixed saturation magnetisation *M*_sat_. Due to their small size (diameters are up to 100 nm), mNPs are considered to be the superparamagnetic ones.^39^ The gradient of the magnetic field of the external magnetic system (consisting of permanent magnets) in simulation is given by the speed of displacement *V⃑*_0_ of LmNPs relative to SmNPs. The direction of the magnetic field gradient (*i.e.*, direction of the relative mNP motion) may not coincide with the direction of the magnetic flux density *B⃑*_0_, because the magnetic fields created by the magnetic system with a large magnetic field gradient are complex (magnets with a spatially rotating magnetization like the Halbach array of permanent magnets^38^) and the direction of the gradient often does not coincide with the direction of the magnetic fields. It is assumed that the gradient of magnetic fields is large enough to give LmNPs a motion speed of 1 to 10 microns per second relative to the SmNP motion.

**Fig. 3:**
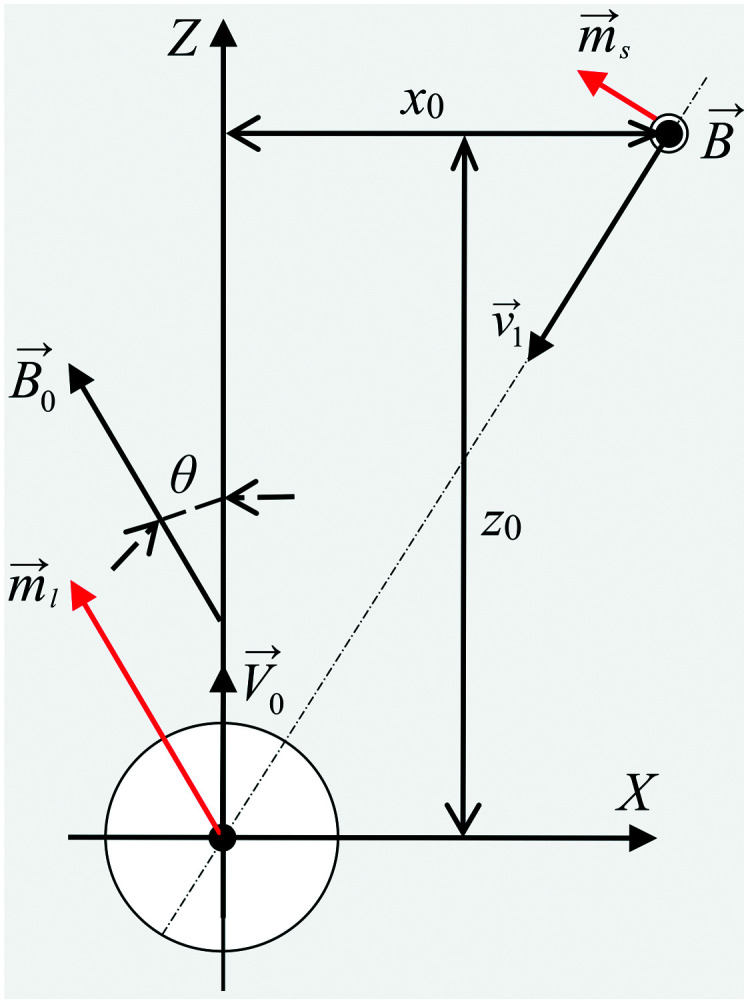
Scheme of the interaction of large and small mNPs in a strong magnetic field gradient.

Due to the relatively small size of the mNPs considered in the analysis (diameters are up to 100 nm), the magnetic field and its gradient across each mNP can be considered as homogeneous. Therefore, the superparamagnetic mNPs will be uniformly magnetized with an orientation directed along the magnetic field. The ferrimagnetic nanoparticles will also be oriented so that the resulting magnetic moment will be directed along the magnetic field. In general, magnetic flux density *B⃑* created by a uniformly magnetized spherical nanoparticle having radius *R* outside the nanoparticle can be written as1
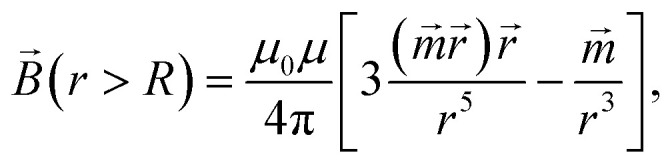
where *μ*_0_ and *μ* are the relative magnetic permeabilities of vacuum and the fluid medium, respectively; 
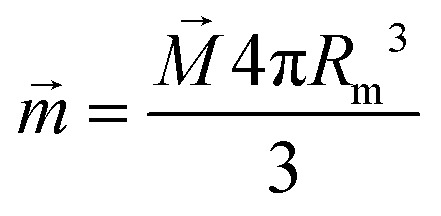
 is the magnetic moment of the nanoparticle (see Fig. 3, where *m⃑*_s_ stands for the magnetic moment of a SmNP and *m⃑*_l_ stands for the magnetic moment of an LmNP), *r⃑* is the radius-vector from the centre of the nanoparticle to the point of the magnetic field determination, *R*_m_ is the radius of the mNP magnetic core (we consider the general case of a core–shell nanoparticle having an additional upper layer, *e.g.* a plasmonic one – in such a case, radius *R* is not equal to the radius *R*_m_), *M⃑* is the magnetization of nanoparticles.

For the superparamagnetic spherical nanoparticles, bulk magnetization *M⃑* in the weak magnetic field approximation is calculated *via* the equation

2 *M⃑* = 3(*μ*_NP_ − *μ*)/[*μ*_0_(*μ*_NP_ + 2*μ*)]*B⃑*_ext_,

where *μ*_NP_ is the relative magnetic permeability of an mNP, *μ* is the relative magnetic permeability of the colloidal fluid medium (for biofluids with sufficient accuracy *μ* = 1), and *B⃑*_ext_ is the flux density of the external magnetic field acting on the mNP (
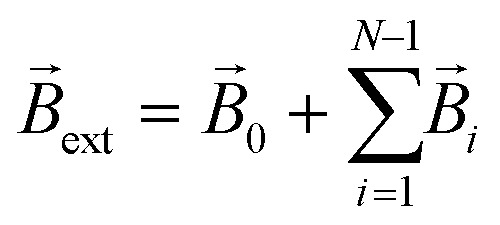
, where *B⃑*_*i*_ stands for the magnetic flux density created by the *i*-th mNP, and *N* is the total number of mNPs). In a strong magnetic field (in the case of the magnetic saturation), bulk magnetization *M⃑* can be calculated with the following equation instead:

3 *M⃑* = *M*_sat_*B⃑*_ext_/|*B⃑*_ext_|.

For a large mNP, the external magnetic flux density *B⃑*_ext_ can be written sufficiently for calculation accuracy as the magnetic flux density of the magnetic system *B⃑*_0_ only, *i.e.*

4 *B⃑*_ext_ = *B⃑*_0_,

where *B⃑*_0_ is magnetic flux density created by the permanent magnets. This assumption does not take into account the magnetic field of small mNPs, because this field is localised in the area much smaller than the size of large nanoparticles, which therefore will have a little effect on the overall magnetization of large mNPs, especially for distances greater than the radius of SmNPs (*i.e.*, tens of nanometres). For an SmNP, the external magnetic field is the sum of the permanent magnet system flux density *B⃑*_0_ and the magnetic flux density *B⃑*_l_ of the nearest LmNP (taking into account only the field of the nearest LmNP and neglecting all other LmNPs due to low concentration of the LmNPs in the solution):5
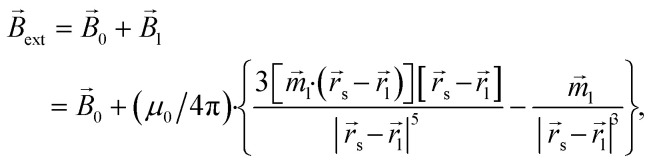
where *r⃑*_s_ and *r⃑*_l_ are the radius-vectors (coordinates) of small and large NPs, respectively.

Force *F⃑* acting on a small mNP in the case of a weak magnetic field (unsaturated mNP magnetisation) is defined as6
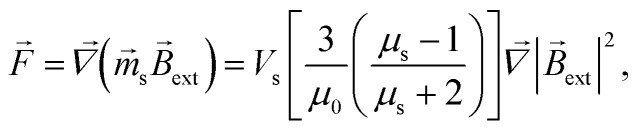
where *μ*_s_ and *V*_s_ are the relative magnetic permeability of the ferrimagnetic material and the volume of the small mNP, respectively. For the unsaturated magnetisation of an SmNP, the volume magnetisation *M⃑*_s_ and the magnetic moment *m⃑*_s_ can be written as7a
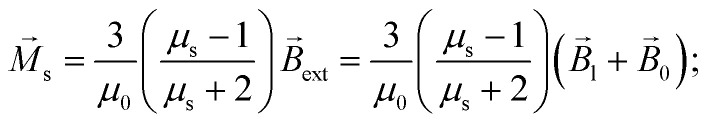
7b
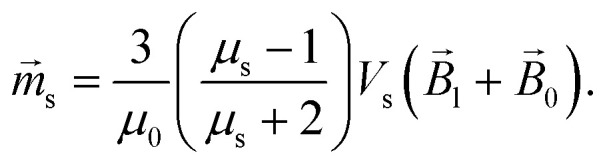
The energy *E* and the force *F⃑* of interaction of two nanoparticles can be written therefore as8a
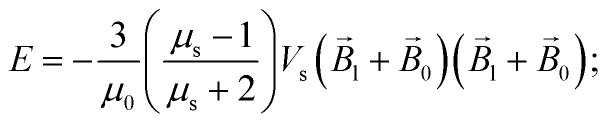
8b
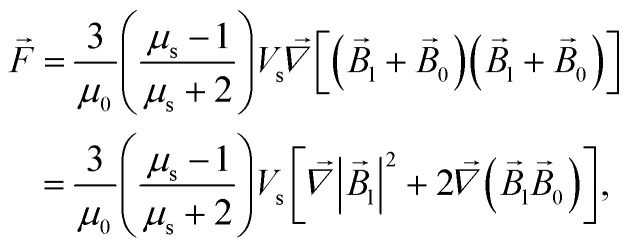
where it is taken into account that the magnetic field gradient of the system of permanent magnets is weak enough not to be able to affect the trajectory of the small nanoparticles, and 
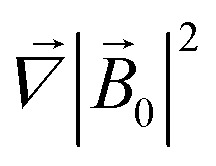
 is therefore neglected. The squared magnetic flux density |*B⃑*_l_|^2^ of the magnetic field of a LmNP and its gradient can be written as9
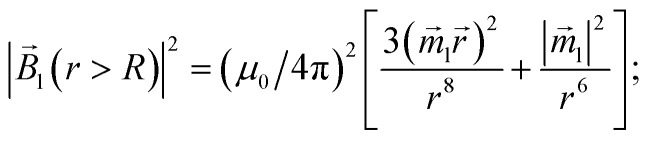
10
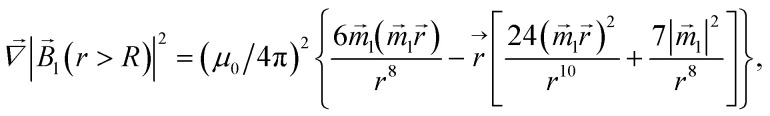
where, hereinafter, the following notation is used: *r⃑* = *r⃑*_s_ − *r⃑*_l_; *r* = |*r⃑*|. The interaction force *F⃑* between nanoparticles depends on the distance between the nanoparticles and their orientation relative to each other. It is assumed that the magnetic field *B⃑*_0_ of permanent magnets is collinear to the plane formed by the displacement vector *V⃑*_0_ of the large nanoparticles and the radius-vector *r⃑* connecting the nanoparticle centres. Furthermore, the Cartesian system with an *XZ* plane parallel to this plane is chosen (see Fig. 3). In this way, all events are considered to occur in this *XZ* plane. This simplification does not principally affect the simulation results because the strength of the interaction is determined by the gradient of the dipole interaction which has the azimuthal symmetry. Under the made assumptions, the components of the gradient of the external magnetic flux density *B⃑*_ext_ in the region of a small mNP will also lie down in the *XZ* plane and, using eqn (5), (9) and (10), can be written as11
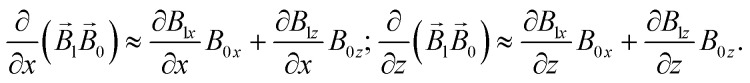
12
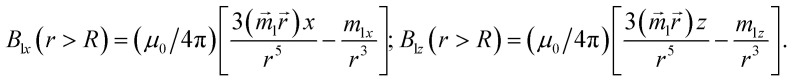
13a
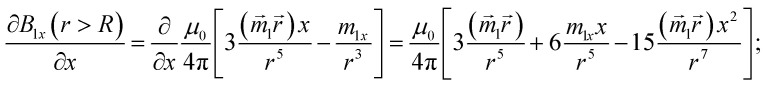
13b
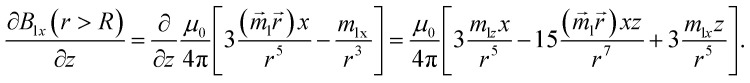
14
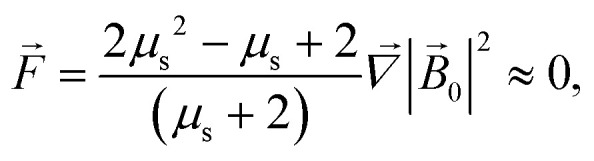
where *B*_l*x*_, *B*_0*x*_ and *B*_l*z*_, *B*_0*z*_ stand for *x*-th and *z*-th components of the magnetic flux density, respectively, created by a nearby LmNP and the system of permanent magnets.

For the strong external field and saturated magnetization, the force acting on an SmNP can be written as15
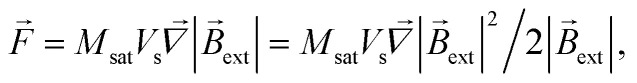
and the gradient of the magnetic flux density for this case is easy to calculate through the gradient of the squared magnetic flux density as16
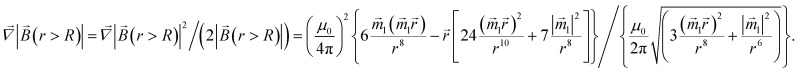


The equation for the relative velocity of mNPs, given the dominance of viscosity forces over inertia, can be written as

17 6π*ηR*_l_·d*r⃑*/d*t* = *F⃑*,

where *η* is the viscosity of the fluid and *R*_l_ is the radius of the LmNP. From the formulas given above, one can see that the interaction of two mNPs consists of two parts. The first part is the interaction between two magnetic dipoles created by the LmNP and SmNP. It is a near-field interaction because it decreases rapidly with the distance *R*_l_^6^/*r*^7^, where *r* is the distance between the LmNP and SmNP, and *R*_l_ is the radius of LmNP. The second part of the interaction is caused by the changes in the intensity of the magnetic field due to the interference of the external field and the LmNP field and the respective vector summation. This interaction spreads over a greater distance as it decreases as *R*_l_^3^/*r*^4^ with the increase of the distance between the LmNP and SmNP, which is much slower than the pure magnetic field of the LmNP.

When the distance between the centres of the mNPs is slightly larger than the diameter of the large mNP, the magnitude of the magnetic field and hence the orientation of the small mNP are mainly determined by the external magnetic field. However, in the immediate vicinity of two mNPs in weak external magnetic fields (unsaturated magnetization), the magnetic field of a LmNP dominates due to almost threefold amplification of the external magnetic field by the LmNP. In intense external magnetic fields, when magnetic saturation is achieved, regardless of the distance between the mNPs, the magnetization is mainly determined by the external field and is weakly dependent on the position of the LmNPs. Looking ahead, simulations show that without the interference term, due to a purely dipole interaction, such as in the case of two ferrimagnetic nanoparticles, effective capture of nanoparticles can only occur at very short distances, when the SmNP approaches the LmNP to a distance of about one LmNP diameter. For the simulation, only the external radius *R*_s_ of small mNPs, external radius *R*_l_ of large mNPs, radius *R*_lm_ of the magnetic part of the LmNPs, radius *R*_sm_ of the magnetic part of the SmNPs, and the parameters of the magnetic material that the mNPs are made of are important. Since small mNPs are considered to be the not plasmonic ones, we will assume that they are completely magnetic nanoparticles, and therefore *R*_s_ = *R*_sm_.

Let us estimate the velocities moving at which small and large nanoparticles will be able to “adhere” to each other due to the dipole interaction. The solution viscosity (not the inertia) determines the displacement velocity, which is proportional to the squared radius of the particle. The LmNP radius is more than 10 times higher than that of the SmNP. Therefore, the displacement velocity of the small nanoparticle due to the SmNP–LmNP interaction will be much higher than that of the large one, and it can be assumed that only small nanoparticles are displaced during the interaction. Based on the proposed mathematical model, a program for the SmNP trajectory simulation when a large nanoparticle passes at a certain distance was developed using the C++ programming language. Simulation results will be presented in the following section.

## Simulation results

The magnetic properties of the spherical mNPs used in the simulation were taken from ref. 28 (see Table 1).

**Table 1:** Linear sizes and magnetic properties of mNPs used in the simulation of the interaction of mNPs in colloidal solution

	*R*_m_ (magnetic core radius) [nm]	*R* (mNP outer radius) [nm]	*μ*_NP_ (mNP relative permeability)	*M*_s_ (mNP saturation magnetization) [A m^−1^]
SmNP	2.5	2.5	400	2.25 × 10^5^
LmNP	25.0	35.0	500	3.00 × 10^5^

The magnetic system with the permanent magnets can create a maximum magnetic flux density |*B⃑*_0_| of about 1.00 T. Therefore, the simulation was carried out for the external magnetic field |*B⃑*_0_| in the range of 0.05–1.00 T. We used the weak force model if the magnetization due to the NP permeability, given in Table 1, is lower than the saturation magnetization and the strong field model in the other case. The simulation scheme is shown in the previous section in Fig. 3. Analysis was performed for different distances *x*_0_ of the trajectory of a large nanoparticle from the initial position of a small nanoparticle (on the graphs below, this distance is normalized to the radius *R* of an LmNP. Note that hereinafter, we mark the radius of LmNPs by radius *R*, instead of *R*_l_, because we will not use the notation *R*_s_ of the radius of SmNPs.).

The direction of the magnetic field may change rapidly depending on the position of the measuring point relative to the external magnets. In this way, the inclination angle of the magnetic field relative to its gradient, angle *θ* (see Fig. 3), can vary significantly depending on where the magnetic system trajectories of large and small mNPs intersect. Therefore, the simulations were performed for the following angle *θ* values: 0°, 52°, and 90°. In the simulation, it was assumed that the LmNP moves along the *Z*-axis with the initial coordinates of (*X*, *Y*, *Z*) = (0, 0, −10*R*), and the initial coordinates of the SmNP are (*x*_0_, 0, 0).

**Fig. 4:**
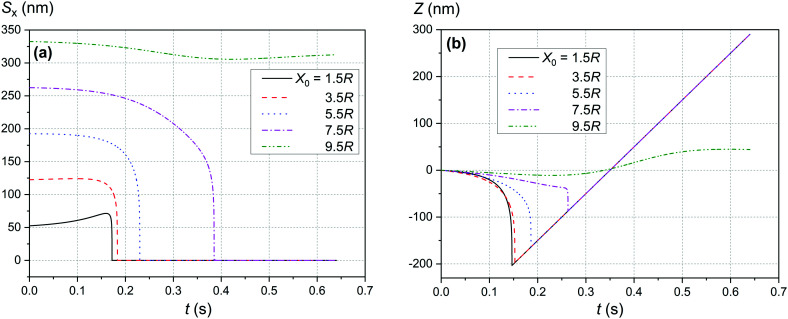
Movement trajectory of the SmNP near the LmNP moving with a speed of 1 μm s^−1^ in a weak magnetic field *B* of 0.10 T for different distances *x*_0_: (a) distance *S*_*x*_ between nanoparticles in the horizontal direction along the *x*-axis and (b) its movement in the vertical direction along the *z*-axis; *θ* = 52°.

**Fig. 5:**
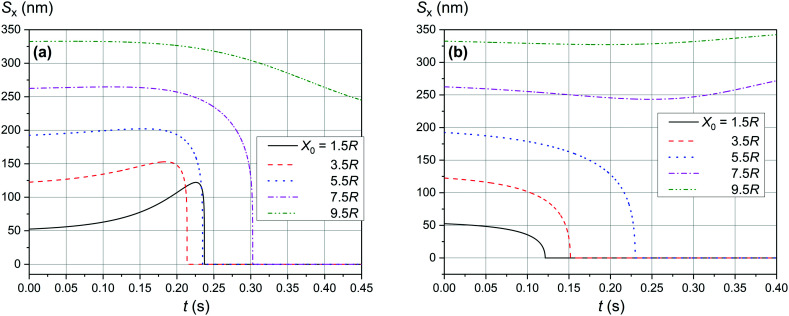
Convergence between mNPs in the horizontal direction (along the *x*-axis) in a weak magnetic field *B* of 0.10 T in the case of a large nanoparticle speed of 1 μm s^−1^ for different distances *x*_0_ of a SmNP to the LmNP trajectory: (a) *θ* = 90°; (b) *θ* = 0°.

**Fig. 6:**
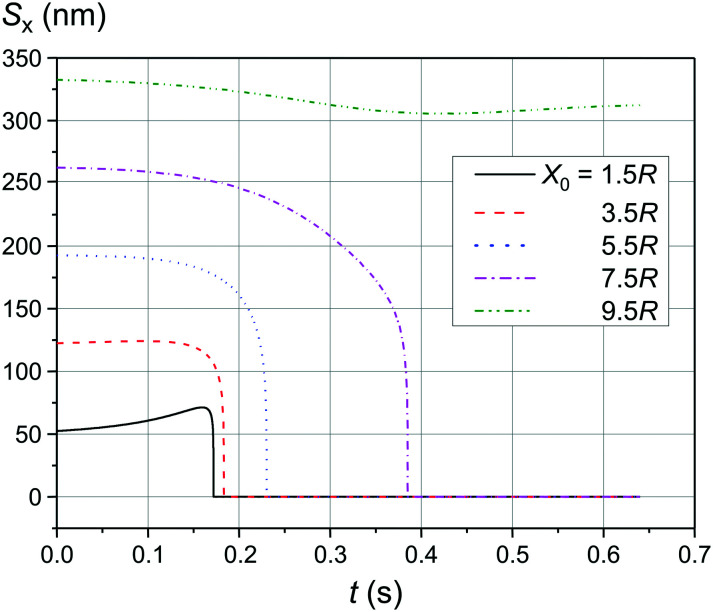
Trajectories convergence of mNPs in the horizontal direction (along the *x*-axis) in a weak magnetic field *B* of 0.05 T in the case of large nanoparticle speed of 1 μm s^−1^ for different distances *x*_0_ of a small mNP to the trajectory of the large mNP; *θ* = 52°.

**Fig. 7:**
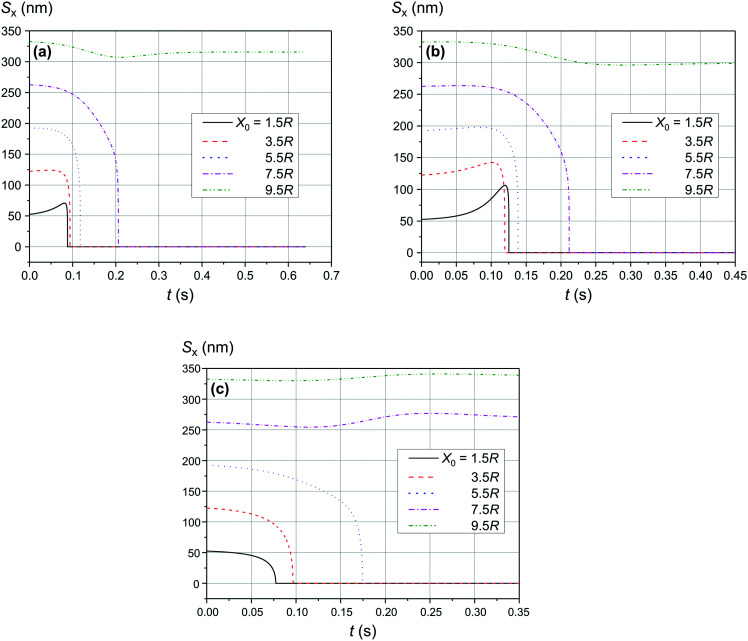
Trajectories convergence of mNPs in the horizontal direction (along the *x*-axis) in a weak magnetic field *B* of 0.10 T in the case of large nanoparticle speed of 2 μm s^−1^ for different distances *x*_0_ of a small mNP to the trajectory of the large mNP: (a) *θ* = 52°, (b) *θ* = 90°, and (c) *θ* = 0°.

**Fig. 8:**
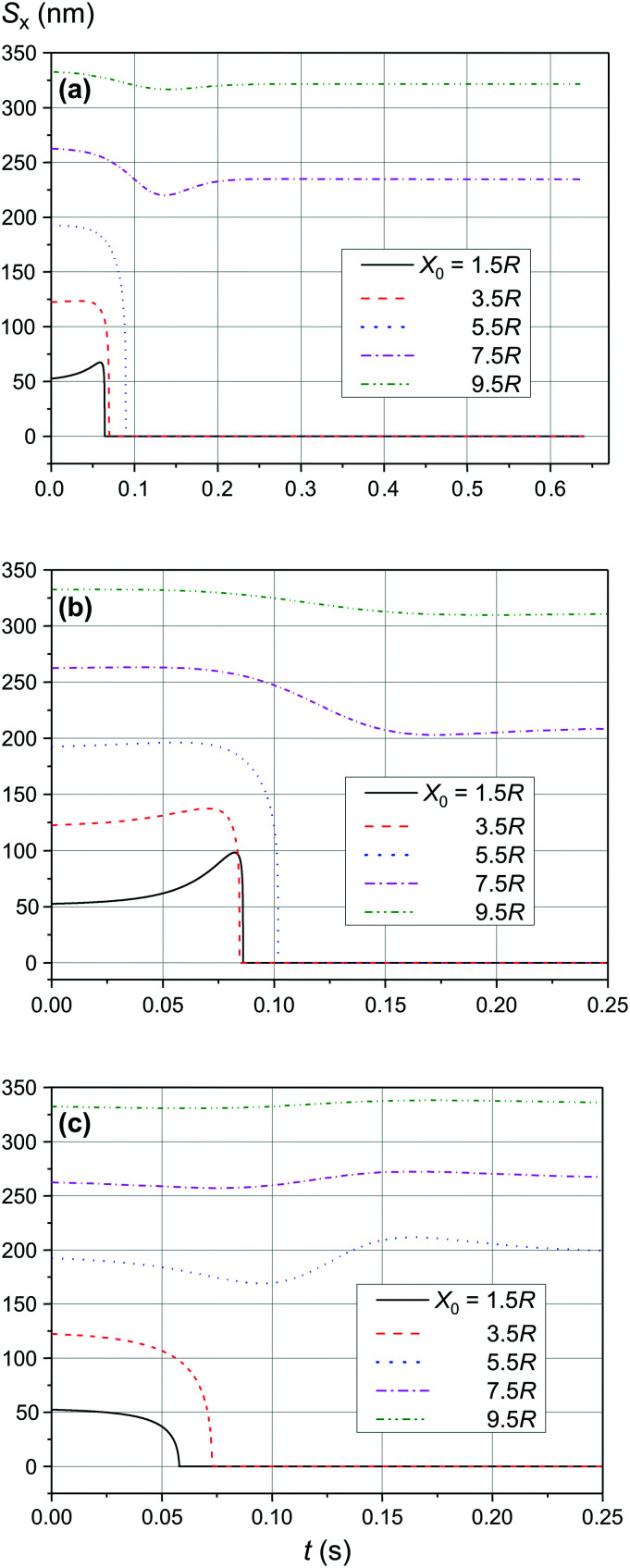
Trajectories convergence of mNPs in the horizontal direction (along the *x*-axis) in a weak magnetic field *B* of 0.10 T in the case of large nanoparticle speed of 3 μm s^−1^ for different distances *x*_0_ of a small mNP to the trajectory of the large mNP: (a) *θ* = 52°, (b) *θ* = 90°, and (c) *θ* = 0°.

**Fig. 9:**
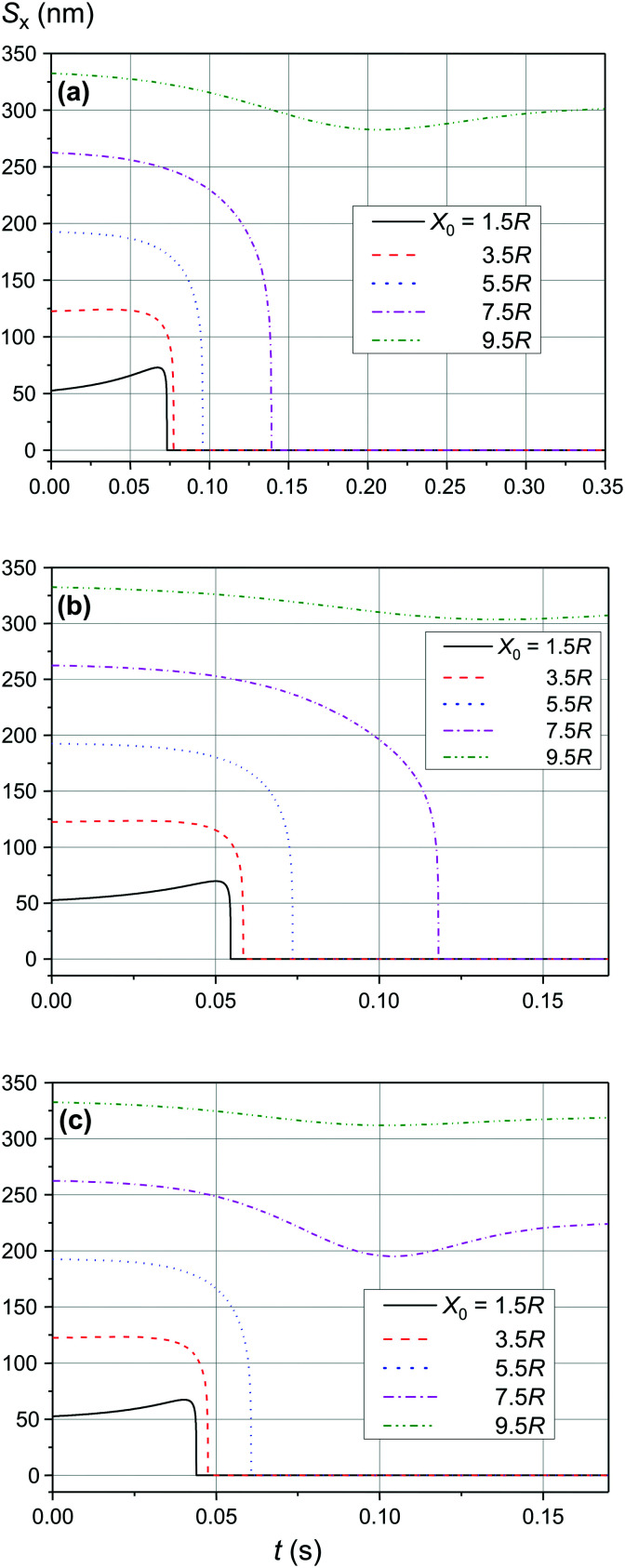
Trajectories convergence of mNPs in the horizontal direction (along the *x*-axis) in the magnetic field *B* of 0.50 T for different distances *x*_0_ of a small mNP to the trajectory of the large mNP; *θ* = 52°: large nanoparticle speed of (a) 2 μm s^−1^, (b) 3 μm s^−1^, and (c) 4 μm s^−1^.

**Fig. 10:**
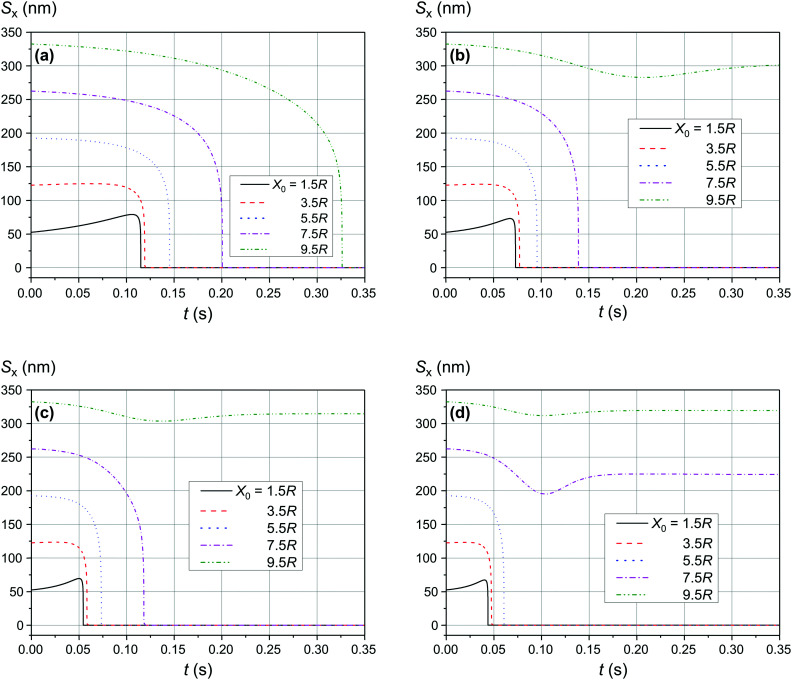
Trajectories convergence of mNPs in the horizontal direction (along the *x*-axis) in the magnetic field *B* of 1.00 T for different distances *x*_0_ of a small mNP to the trajectory of the large mNP; *θ* = 52°: large nanoparticle speed of (a) 1 μm s^−1^, (b) 2 μm s^−1^, (c) 3 μm s^−1^, and (d) 4 μm s^−1^.

Fig. 4 and 5 show the motion of the SmNPs during the passage of a LmNP in a weak magnetic flux density *B* of 0.10 T. As shown in Fig. 4, the SmNPs at the initial distances *x*_0_ of up to 7.5*R* from the trajectory of the large nanoparticle gradually converge to the LmNP, followed by a rapid acceleration when the distance between them decreases to the radius *R* of the LmNP, *i.e.*, to the distance when the pure dipole interaction is effective. At large distances, when the pure dipole interaction is not effective yet, slow convergence between mNPs is achieved through the interference term of the magnetic interaction. After the collision, SmNPs are settled on the LmNP. This is clearly seen in Fig. 4(b) where, after the adhesion of a SmNP to the LmNP, the “combined” magnetic nanoparticle moves at the speed of the LmNP. In the following results (Fig. 4–10), the convergence between mNPs is showed as a horizontal distance *S*_*x*_ between mNPs: *S*_*x*_ = *x*_l_ − *x*_s_, where *x*_l_ and *x*_s_ are the *x*-coordinates of large and small mNPs, respectively.

Fig. 6 shows the simulation results of the convergence of two mNPs even at a lower external magnetic field *B* of 0.05 T. The comparison of the data provided in Fig. 4(a) and 6 shows that at low speeds of LmNPs (of the order of 1 μm s^−1^ in a gradient magnetic field), a significant weakening of the magnetic field does not lead to a significant change in the capture efficiency of SmNPs by LmNPs, *i.e.*, it does not lead to a significant change in the adsorption radius (we name by “adsorption radius” the maximum distance of the LmNP from the initial position of the SmNP at which the LmNP can “adsorb” an SmNP).

The comparison of the data in Fig. 4 and 5 with the data in Fig. 6–8 that differ only in the speed of displacement of large nanoparticles (*i.e.*, gradient of the squared magnetic flux density) shows that the efficiency of SmNP adsorption by LmNPs decreases rapidly with increasing speed. Moreover, it is easy to see that they all have a certain dependence on the orientation direction of mNPs – this is due to the fact that the nanoparticle interaction depends on the interference term of the interaction, which is responsible for the slow initial convergence of the two mNPs.

As shown in Fig. 9 and 10, the significant increase of the magnetic field to 0.50 T and 1.00 T does not lead to a significant increase of the adsorption efficiency (= absorption radius) of a small SmNP on the LmNP surface. However, as shown by the magnetic field simulation, the magnetic field gradient of the magnetic system decreases at approximately the same speed as the magnetic field itself with increasing distance to external magnets. Therefore, the drift (displacement) velocity *V⃑*_0_ of the LmNP decreases at the same approximate relative speed as the magnetic field. In this way, the efficiency of adsorption of small mNPs on the surface of large ones will be higher in the region of weak magnetic fields (= small external magnetic field gradient) due to the small speed of displacement of large mNPs in this region. On the other hand, the adsorption efficiency in the regions of strong magnetic field decreases but instead there is a rapid movement to the place of mNP concentration. In other words, the areas with a small gradient of external magnetic field are the areas of efficient SmNP adsorption by the LmNPs (coagulation areas, *i.e.*, areas of adhesion of small mNPs to large ones), and the areas with a large gradient are the areas which provide rapid concentration of LmNPs into the detection area.

## Discussion

**Fig. 11:**
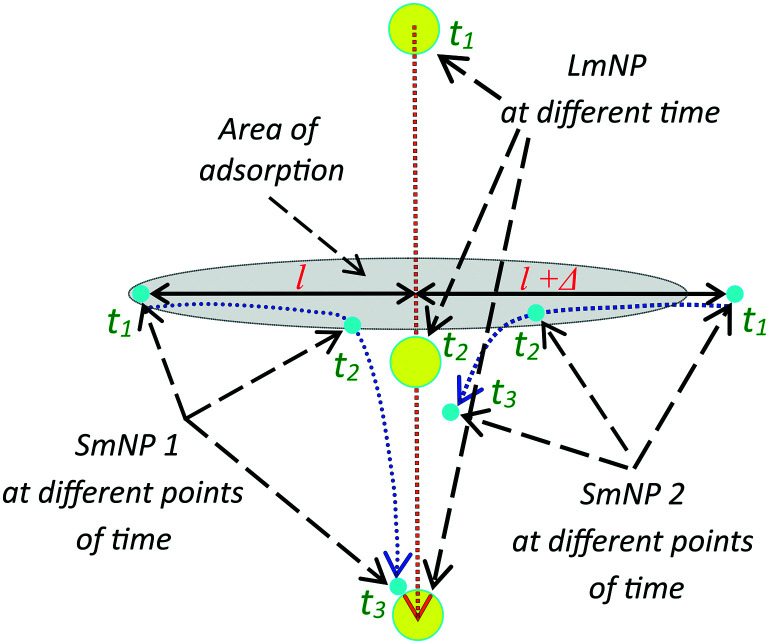
Trajectories of large and two small mNPs in the gradient magnetic field; the mNP positions are shown at three points of time: the initial position of SmNP 1 is in the adsorption area, while SmNP 2 is outside the area.

The magnetic field gradient in the simulation above was described through the speed of the large nanoparticles (LmNPs). The LmNP speed used in simulation was evaluated using the magnetic properties of magnetite or maghemite medium and the magnetic field of millimetre linear size permanent magnets. In the simulation, only the mNPs with the size of *R*_l_ = 35 nm and *R*_s_ = 2.5 nm were used. However, there is a question if these are the optimal sizes and what will happen if one changes the nanoparticle sizes. This question is more interesting for the LmNPs because the SmNP size range is limited by the proposed method. Since the magnetic field force is proportional to the magnetic medium volume *V* (*V* ∼ *R*^3^) and the resistive force of viscosity increases as ∼*R* (inertia forces are small and can be neglected), the speed of the LmNP under the magnetic field should increase as *R*^2^ and the effective time of interaction *τ* of two mNPs decreases as ∼*R*/*R*^2^ = 1/*R*. The gradient of the magnetic field created by large mNPs decreases as ∼1/*R*, which results in the decrease of speed of the SmNP as 1/*R*. Thus, in the same magnetic field, the SmNP will move towards the LmNP during their time of interaction *τ* at distance *l* (see Fig. 11):*l* = *τ* × *v* ∼ 1/*R* × 1/*R* = 1/*R*^2^,where *l* can be considered as a distance at which the SmNP–LmNP interaction is effective enough for the SmNP to be adsorbed by the LmNP during their relative motion, *v* is an average speed of the SmNP motion towards the LmNP during their effective interaction, and *τ* is the time of their effective interaction (before the moment of the adsorption of the SmNP on the surface of the LmNP). In other words, by increasing large nanoparticles size, one decreases the area of the effective small nanoparticle adsorption as 1/*R*^4^ (see Fig. 11). Thus, by increasing the LmNP size and using the same magnetic system, one gets strong decrease in the adsorption efficiency of the SmNPs. However, it is not difficult to see that we can solve the problem of more effective adsorption by decreasing the magnetic field force resulting in the proportional decrease of large nanoparticle speed. Equal adsorption area can be obtained using a system with the same squared magnetic flux density but decreased magnetic field gradient on the scale of *R*^2^. However, in this case we will have the same system productivity due to the same LmNP speed ∼*R*^2^/*R*^2^ but at increased LmNP size, which is not good for the signal detection.

Therefore, for an effective small nanoparticle adsorption by the large ones with a high ratio of mass of the large mNPs to the small ones, the size of the large mNPs has to be as small as possible. However, this requires a substantial increase of the magnetic field gradient (as 1/*R*^2^) to maintain the large mNP speed value (*i.e.*, to keep the same system productivity). However, the gradient of the magnetic field of a permanent magnet system cannot be increased infinitely – this is the obstacle that inspired us to propose a method with two sorts of magnetic nanoparticles utilization. Thus, for available magnetic system with one magnet linear size of ∼1 mm, the diameter of large mNPs should be at least several tens of nanometres.

It is also interesting to mention that at some points in time and in some cases the distance between the two particles starts to increase again (after a period of the distance decreasing), as one can see in, *e.g.*, Fig. 4a and 5a (black curves, *x*_0_ = 1.5*R*). This behaviour can be explained as follows: when small and large nanoparticles are in the same plane orthogonal to the magnetic field but are not close enough, both of them are therefore aligned along the magnetic field of the external magnetic system; consequently, two magnetic dipoles have the same orientation and repulse each other.

In the considered model, it was assumed that magnetic nanoparticles are aligned with the local magnetic field. This assumption is true in the case when magnetic energy of the interaction of dipole with the magnetic field is significantly larger than the thermal energy. For small magnetic nanoparticles (they are more critical to this approximation) with a radius of *R* = 2.5 nm and with a magnetization *M* of 2 × 10^5^ A m^−1^ in a strong magnetic field *B* of 1 T, the energy ratio can be estimated as0.5*kT*/*mB* = 0.5*kT*/[*M*(4/3)π*r*^3^*B*] = 0.5 × 1.38 × 10^−23^ × 300/[2 × 10^5^ × (4/3)π(2.5 × 10^−9^)^3^ × 1] ≈ 0.15 < 1.This approximation is thus relatively accurate for a strong magnetic field. However, for a weaker magnetic field, the magnetic interaction will be smaller, and for a more accurate calculation, statistical averaging is needed to obtain the average magnetic moment for small magnetic nanoparticles at every new position of the relatively large ones.

In the approximation used for the two-particle interaction analysis, we did not take into account the Brownian motion of the magnetic nanoparticles. Our estimation has shown that during the interaction of the two particles (around 0.1 s), the average small particle shift is about a few microns which may significantly decrease the interaction efficiency of the two particles. On the other hand, we also did not consider small particles dragging by the fluid near the fast-moving LmNPs, which may improve the capture efficiency.^42^ Therefore, the obtained results are the first step toward a new method of the biomarker detection, and we are planning to improve our model in future research by taking into account all the factors mentioned above and, thus, to increase the accuracy of the method evaluation.

## Conclusions

To conclude, in the present paper we proposed to use two sorts of magnetic nanoparticles (mNPs) as a novel non-toxic approach of diagnostic biomarker extraction and concentration from biofluids. In the framework of the method, firstly, small mNPs (with a diameter of 2–5 nm) bearing molecular traps for biomarkers on their surface are used as an agent to be injected into a patient's body and for collecting all specific biomarkers with high sensitivity and selectivity by circulating them in the body.^40,41^ Due to their small size, these mNPs do not lead to the body's intoxication and can be easily released after circulation in the form of a biofluid solution such as urine or saliva. From this perspective, the proposed method benefits from using small (2–5 nm) mNPs circulating in the human body when compared with other existing techniques such as manipulation of only large (200–500 nm) mNPs in the magnetic field^18–20^ which does not allow their injection into the human body due to the high risk of body intoxication. Secondly, large mNPs (with a diameter of tens of nanometres) are afterwards injected into the biofluid solution, and the solution is mixed to obtain a homogeneous distribution. Large mNPs are core–shell nanoparticles in which the shell layer is plasmonic and serves for the plasmonically-enhanced detection of the biomarkers.^14–17^ As a final step, a thin flow of the obtained homogeneous biofluid solution is placed under a strong gradient magnetic field created by an array of external permanent magnets.

The results of the theoretical analysis and mathematical simulation revealed that, depending on the external magnetic field flux density and gradient, small mNPs can be effectively trapped (“adsorbed”) by large mNPs at distances of up to 7.5*R*, where *R* is the radius of large mNPs, *i.e.* up to *ca.* 260 nm, and afterwards be directed to the place of their optical interrogation. Such relatively big adsorption radius is possible due to the existence of an interference term in the nanoparticle magnetic interaction, while without this term – due to a pure dipole interaction (such as in the case of two ferrimagnetic nanoparticles) – effective capture of small mNPs is limited to the distances of one large mNP diameter.

The reported results aim to provide guidelines for the development of a new subclass of biosensors and a non-toxic human body diagnostic approach with enhanced sensitivity and selectivity. Moreover, the provided theoretical results may give new insights for the development of other types of sensing technologies, *e.g.*, the ones utilizing self-assembly to organize particles in the desired architectures,^43^ or may be helpful in the microfluidic particle sorting technologies.^44^

## Author contributions

All authors have given approval to the final version of the paper.

## Conflicts of interest

There are no conflicts to declare.

## Supplementary Material

SD-001-D2SD00078D-s001
