# Supplementary material for: Combined small and large magnetic nanoparticle extraction and concentration from biofluids for non-toxic detection of biomarkers
Source: Sens Diagn. 2022 Jun 9;1(4):829–40. doi: 10.1039/d2sd00078d (PMC9280443; doi:10.1039/d2sd00078d)
Supplement: SD-001-D2SD00078D-s001 [file SD-001-D2SD00078D-s001.pdf]

Electronic Supplementary Material (ESI) for Sensors & Diagnostics.
This journal is © The Royal Society of Chemistry 2022

Electronic Supplementary Information

Combined small and large magnetic nanoparticles extraction and concentration from biofluids for non-toxic detection of biomarkers

Anatoliy S. Lapchuk,[1*] Ivan V. Gorbov[1], Alexander V. Prygun[1], Iryna V. Balagura[1], and Yevhenii M. Morozov[1,2*]

[1]Department of Optical Engineering, Institute for Information Recording of NAS of Ukraine, 03113 Kyiv, Ukraine
[2]Biosensor Technologies, AIT-Austrian Institute of Technology, 3430 Tulln, Austria
[*]Corresponding authors:
Anatoliy S. Lapchuk (email: alapchuk@yahoo.com)
Yevhenii M. Morozov (email: Yevhenii.Morozov@ait.ac.at)

List of the mathematical notations

$\vec{V}_0$– displacement speed of the LmNPs relatively to SmNPs (*NB*! At the same time, this quantity determines the gradient of the external magnetic field).

μ_0 – relative magnetic permeability of the vacuum.

μ – relative magnetic permeability of the biofluid medium.

μ_{NP} – (fixed) relative magnetic permeability of the mNPs (regardless of the mNP's sort).

μ_s – relative magnetic permeability of the SmNPs.

M_{sat} – (fixed) saturation magnetisation of the mNPs (regardless of the mNP's sort).

$\vec{M}$ – magnetisation of an mNP (regardless of the mNP's sort).

$\vec{B}_0$ – magnetic flux density of the external system of permanent magnets.

$\vec{B}$ – magnetic flux density created by a uniformly magnetized spherical mNP.

$\vec{B}_{ext}$ – external magnetic flux density.

$\vec{B}_l$ – magnetic flux density of an LmNP nearest to the considered SmNP.

$\vec{v}_i$ – velocities of the small mNPs ($i = 1, 2, 3, \ldots, n$; where n – number of small NPs).

R – radius of an mNP (regardless of the mNP's sort).

R_s – (external) radius of the SmNPs.

R_l – (external) radius of the LmNPs.

R_m – radius of an mNP's magnetic core (regardless of the mNP's sort).

R_{sm} – radius of the magnetic core the SmNPs.

R_{lm} – radius of the magnetic core the LmNPs.

$\vec{r}$ – radius-vector from the centre of an NP to the point of the magnetic field determination.

$\vec{r}_s$ – radius-vector (coordinates) of an SmNP.

$\vec{r}_l$ – radius-vector (coordinates) of an LmNP.

$\vec{m}$ – magnetic moment of an mNP (regardless of the mNP's sort).

$\vec{m}_s$ – magnetic moment of an SmNP.

$\vec{m}_l$ – magnetic moment of an LmNP.

π – the number π.

V_s – volume of an SmNP.

$\vec{F}$ – force of the mNPs interaction.

E – energy of the mNPs interaction.

$\vec{\nabla}$ – del (nabla) operator.
